# Strictures

**Published:** 1849-01

**Authors:** Wm. A. Pease

**Affiliations:** Dentist.—Dayton, O


### STRICTURES

### ON AN ARTICLE IN THE AUGUST NUMBER OF THE LONDON LANCET, BY J. B. MITCHELL, M. D., &C., LONDON.

BY WM. A. PEASE, DENTIST.—*Dayton, O.*

Often the dental surgeon, while perusing the writings of his brethren in the medical profession, has his feelings and sensibilities wounded by strictures on the practice of his particular branch of the profession, by men whose opinions he otherwise, in common with other medical men, respects; and these opinions are often published simply on the authority of an M. D., and a general knowledge of the pathology and the anatomy of the parts, acquired while pursuing their studies at college, often consisting of but little more than learning the names of the teeth, and curative treatment in the use and application of a rusty turnkey. On the intelligent practitioner, these publications exert no other influence than pity for the ignorance and wonderful complacency of the writer; but not so on the public and those practitioners who pay but little attention to the subject—who are expected, and do, take their cue from these writings, and, in their turn, iterate and reiterate them to their immediate friends, till finally the dentist is surrounded by a wall of prejudice, fortified by the incontrovertible authority of Dr. M., backed by our educated author. Nor is this influence so small as might at first view be imagined—the physician coming in contact and holding daily communication with all classes of society, and being universally considered as having been educated with direct reference to healing all the diseases of the system; and if at all successful, he has acquired the confidence of the community, especially if his popularity is sustained by any claims to an author, exerts a great influence, and is considered the referee on all occasions, and on all diseases. All must admit the dentist has enough to contend with, in combating the whims and ignorance of his patients, without having them increased by the physician; and as the object of both is the same—the promotion of the health and happiness of their species, by removing and preventing pain and disease—they ought to possess confidence in, and mutually assist each other.

The immediate occasion of these and following remarks, although I have frequently seen articles by medical writers equally objectionable, is an article in the last (August) number of the London Lancet, by J. B. Mitchell, M. D., &c., London, which commences by saying, "There is no department of practice in which so many pernicious errors are continually permitted to go uncorrected as of dental surgery." He here, says the Editor of the Lancet, refers to the report of a case published in the Lancet of November 24th last, in which the writer states, "I then extracted, &c., and cut across and destroyed the nerves of the four upper incisors." Dr. Mitchell continues, "I am perfectly aware that the latter of these operations is of every day occurrence among dentists, but it is, nevertheless, one that cannot for a moment be defended on scientific grounds." Now the natural progress of dental caries is from decay of the crown to destruction of the pulp, and gradual wasting of the fang, the latter process being uniformly accompanied with more or less of morbid action in the periosteum of the socket. The most painful of these several stages is, beyond doubt, that in which the pulp is affected; but the subsequent stage, involving, as it does, the periosteum, is infinitely more pernicious to the health and well-being of the rest of the mouth. A single quotation from Sir Benjamin Brodie must set this at rest. "The inflammation on which the toothache depends," says that gentleman in one of his clinical lectures, "terminates, as it always does, in the death of the pulp in the tooth. Then the whole tooth dies, and is now like a portion of dead bone, or any foreign substance stuck in the jaw (Med. Gazette, vol. xv., p. 374); operations, therefore, for the artificial destruction of the pulp of a carious tooth, have no higher object in view than the hurrying of the disease through its most painful stage, in order the sooner to induce the more advanced and far more injurious one. They are, in fact, operations for the more speedy production of stumps; their sole aim is to remove pain at the expense of a greater evil. As such, it is evident that they have nothing in common with the operations of general surgery, which have for their object ultimate benefit, though purchased by immediate pain; and it is but natural to suppose that they owe their origin to the urgency of the patient, rather than the suggestions of science. When a tooth is so far gone as to be incapable of restoration without destroying the pulp, the sooner it is extracted the better; for the mouth is sure to suffer ultimately in proportion to the number of dead teeth allowed to remain."

Perfection in the practice of dentistry is not claimed, even as practiced by the best; yet it is claimed, that for the rapid progress in improvement it has made; for the close investigation of its members, and the application to it of science on scientific principles; the comfort produced and amount of suffering saved, it can, and justly, claim an equality with any department of the healing art. And notwithstanding some medical societies have refused to admit as members some who have chosen dentistry as the particular field of their labors, the public fully appreciate their worth, and will not fail ultimately to vindicate their cause. What in the present case calls down the malediction of Dr. Mitchell, is the destruction of nerves in carious teeth, in which they have been exposed and become troublesome. Although I am no advocate for, and have never practiced, the destruction of nerves, in the particular manner of which he speaks, and believe it is less effectual, and more likely to be productive of "pernicious" consequences, besides being little practiced in this country; still the injury of which he complains results from the destruction, not from the manner, and it is contended, that in a tooth in which caries has not so far progressed as to disorganize and destroy the life of the structure, but has penetrated directly to the nerve, without extensively burrowing under the enamel, when the pulp cannot be caped, (and it seldom can,) and when the gum and investing membranes are healthy without alveolar absorption—in short, in a tooth which, had the nerve not been exposed, would be considered safe for plugging, that it is not only judicious, but good practice, to destroy the nerve, or at least that portion exposed, (for this can often be done,) and at a proper time fill it.* True, as practiced by some, arsenical preparations may be used to excess, or suffered to remain too long in the tooth, by which the investing membrane may become diseased or destroyed, and thus render the tooth valueless, and a source of irritation and trouble; but in the careful application of this remedy even, much suffering is often prevented, a valuable tooth is saved for years, if not for life, to subserve a useful purpose, and that too in cases where, from the previous loss of teeth, the tooth could be but illy spared, either for mastication, or, as it often happens, is necessary to sustain artificial teeth. To those who have many patients from the country, these remarks will be obvious; there, there is not that attention paid to the teeth as in the city; often the first intimation the person has that his teeth are defective, while masticating some hard substance, the crown of the tooth crushes in and comes in contact with the nerve, causing excruciating pain; yet even these cases, extreme as they are, can often be saved with little or no pain, and made to subserve a valuable purpose for years. The error under which Dr. Mitchell labors is, that by the destruction of the pulp, the tooth must necessarily die, and become a foreign extraneous substance, "like any other portion of dead bone stuck in the jaw," and consequently a constant source of irritation; this, though at times true, is not a universal, necessary, or common consequence, where the operation has been properly performed. The destruction of the nerve, which ought always, if practicable, to be avoided, while it diminishes, does not entirely interrupt the circulation through the tooth, which is supplied by the periosteum in sufficient quantities, to keep it healthy and of a natural color, and retain it firmly in its place. A dead tooth is obvious to the eye by its change of color, and to the touch by oscillation; besides it is not sensitive, like other teeth.

I have had occasion in teeth, in which I had previously

* We have our doubts as to the correctness of the above. We have always supposed that any application which might destroy the most depending part, would not fail to ultimately destroy the whole to the apex of the fang.—CIN. ED.

destroyed the nerve and filled, subsequently to plug another cavity, and have found them as sensitive to the touch of the instrument as other teeth.* There is in my own mouth a tooth which some four years ago suddenly became painful; upon examination, it was found that a cavity that had been plugged several years previous had a defect, through which the juices of the mouth had access to the cavity, and occasioned decay, till the nerve was exposed. The plug was removed, and applications employed to allay the inflammation, which resulted in the death of the nerve. Subsequently the tooth was re-filled, and, to this day, is as good and useful as any in my mouth, occasioning no inconvenience; and being a superior central incisor, it is exposed to view, yet the closest scrutiny can detect no difference in its appearance from the adjoining healthy teeth. According to Dr. Mitchell, the practice was a very questionable one, having no higher object in view than the accomplishment of a present good at the expense of a great future evil—in fact, an operation for the speedy production of a stump, and the only good practice would have been the immediate extraction of the tooth, and consequently the substitution of an artificial one—a poor substitute at the best, and only an approximation in utility and appearance to a natural one.

His theory would also prohibit the insertion of pivot teeth on healthy roots, which all dentists of experience and observation know are far superior to plate in some instances, especially for a single tooth. It is not to be inferred from the above observations that the indiscriminate destruction of nerves is advocated. Where a tooth cannot be preserved by plugging, it ought unquestionably to be removed; still, there are times when even this ought to be deferred, and the pain allayed, or the

* When the nerve has been destroyed, we have never found vitality in the crown. In the root, as you approach the outer surface or periosteum, sensation is often apparent; hence, as Dr. Pease remarks, we have cause to regard Dr. Mitchell's assumption as erroneous, and that they are not always as "dead bones stuck in the jaw." Many of our best dentists doubt the practicability of destroying the nerve of a tooth and then filling it. In teeth of single fangs we think this, however, can often be done to advantage. Teeth with more roots we have generally found more apt to cause suffering. When expedient, we prefer cutting out the nerve, just as is generally done preparatory to the insertion of a pivot tooth.—CIN. ED.

nerve destroyed, as in some peculiar habits and idiosyncrasies, in pregnancy or debilitated constitutions, when much nervous excitement is apprehended. Who has not seen a delicate female, perhaps in some of the last months of pregnancy, when her nervous system is peculiarly sensitive to outward impressions, and when, from the febrile condition of her system, caries has been induced, (a frequent acompaniment,) suffering for days and weeks with that most unbearable of all pains, the toothache; and when, at last, from protracted pain and sleepless nights, she is completely exhausted, and compelled to place herself in an operating chair, what operator has not witnessed with alarm her great nervous agitation, and feared the most fatal consequences might result, if not to the mother, to her unborn offspring? And when informed that what, to her excited imagination, seemed so dreadful an operation, need not be performed—that her teeth can be saved, the pain allayed, and that too immediately, and then witnessed the glow of pleasure and gratitude on her countenance, as she calmly submits to the necessary applications, and experienced a conscious pride, a heartfelt joy, that he has it in his power to alleviate so much distress? These are not imaginative, isolated cases, but they are of frequent, if not daily occurrence; nor confined to families alone, but here, in the west, the periodical return of fever is sure to produce a crop of fresh cavities, which not unfrequently progress so rapidly as to expose the pulp before the patient recovers sufficiently to endure the fatigue of having them plugged. What is to be done? Extract them? In that case, the bilious people and young married women must dread, and justly, the return of the season when they know their systems must be under more or less febrile excitement, and especially the female, whose system is so much more susceptible of febrile impressions, and the long continuance of the action, often on teeth but slightly constituted, the component parts of which are peculiarly adapted to the action of caries. Surely if every tooth that may happen to have the nerve exposed must be sacrificed, she has a sad prospect before her, as she is sure, before she can become the mother of many children, while yet young, to be as toothless as her babe in utero.

